# New species of the genus *Pseudolathra* Casey, 1905 (Coleoptera, Staphylinidae, Paederinae) from the Northwestern District of China

**DOI:** 10.3897/zookeys.1036.60319

**Published:** 2021-05-05

**Authors:** Xiaoyan Li, Yanpeng Cai, Hongzhang Zhou

**Affiliations:** 1 Hebei Key Laboratory of Animal Diversity, College of Life Science, Langfang Normal University, Langfang 065000, China Langfang Normal University Langfang China; 2 Key Laboratory of Zoological Systematics and Evolution, Institute of Zoology, Chinese Academy of Sciences, 1 Beichen West Rd., Chaoyang District, Beijing 100101, China Guizhou University of Traditional Chinese Medicine Guiyang China; 3 Morphological Laboratory, Guizhou University of Traditional Chinese Medicine, Guiyang, 550025, Guizhou, China Institute of Zoology, Chinese Academy of Sciences Beijing China

**Keywords:** Lathrobiini, new species, rove beetles, taxonomy

## Abstract

Two new species of the genus *Pseudolathra* Casey, 1905 from mainland China are reported in this paper, namely *Pseudolathra gansuensis* Li & Zhou, **sp. nov.** and *P. assingi* Li & Zhou, **sp. nov.** This genus is reported for the first time from Gansu Province, Northwest China. Both species are described in detail and supplemented with color plates of normal light photos of the habitus, sternites VII–IX and details of aedeagal structures in different views.

## Introduction

The genus *Pseudolathra* Casey, 1905 (Staphylinidae, Paederinae) is a rove beetle genus in the subtribe Lathrobiina with well-developed hind wings, commonly found during light trapping or in debris (Assing 2012). The genus occurs in all zoogeographic regions, though most of the species have been recorded from the East Palaearctic and Oriental regions and many species have restricted distributions (Rougement 2015; Assing 2018, 2019; Li et al. 2019). So far, there are about 100 species recorded worldwide in the genus. More and more species of the subtribe Lathrobiina will be moved into *Pseudolathra* from related genera within the subtribe, so the exact number of species in the genus is still pending (Li et al 2013; Kocian and Hlaváč 2018).

Based on the most recent knowledge, the genus *Pseudolathra* was composed of 9 Chinese species by 2019 (Herman 2003; Assing 2012, 2013a, b, 2014; Li et al. 2013; Peng et al. 2014; Li et al. 2019), all characterized by the punctures on the forebody, while the middle area of the pronotum is impunctate; the punctures of the elytra are distinctly aligned in rows. Based on material collected recently, two new species from mainland China are described and illustrated here, namely *Pseudolathra gansuensis* Li & Zhou, sp. nov. and *P. assingi* Li & Zhou, sp. nov., both from Gansu province, China, which can be placed in the *P. unicolor* species group in the subgenus Allolathra based on the diagnostic characters of that group (Assing 2012). Thus, there are currently 11 species of *Pseudolathra* known from China. The type specimens are deposited in the Institute of Zoology, Chinese Academy of Sciences, Beijing (IZCAS).

## Material and methods

The dried specimens were softened in hot water at 60 °C for about 8 hours for dissection of the terminalia. The male genitalia were soaked in a 10% KOH solution (30 °C) for about 20–40minutes (depending on the degree of sclerotization). The surrounding soft tissues were immediately removed and the remaining dissected parts are preserved in glycerin in plastic microvials with stoppers, pinned together with the source specimen for subsequent observation and photography. For each species, 3–5 specimens were dissected.

Observations, dissections and measurements were done under a Zeiss SteREO Discovery V20 stereomicroscope. Photos of the habitus, sternites and genitalia were taken with a Zeiss AxioCam MRc 5 camera attached to a Zeiss Axio Zoom V16 Stereo Zoom Microscope. Photos were processed and stacked with the Zen 2012 (Blue version) and Helicon Focus imaging softwares. All specimens listed in the present study were deposited in the Institute of Zoology, Chinese Academy of Sciences, Beijing (**IZCAS**).

The following abbreviations are used in the descriptions:

**AEL** Aedeagus length (length of the aedeagus from apex of dorsal plate to base of aedeagal capsule);

**BL** Body length (measured from anterior margin of labrum to end of abdomen);

**EL** Elytra length (measured from humeral angle to posterior margin);

**EW** Elytra width (width of elytra across the widest part);

**EYL** Eye length (length of eye in dorsal view);

**FL** Forebody length (measured from anterior margin of labrum to posterior margin of elytra);

**HL** Head length (measured from anterior margin of clypeal to posterior constriction);

**HW** Head width (greatest width of head, including eyes);

**PL** Pronotum length (measured from anterior margin to posterior margin);

**POL** Postocular length (measured from posterior margin of eye to posterior constriction of head);

**PW** Pronotum width (greatest width of pronotum).

## Taxonomy

### Pseudolathra gansuensis

Taxon classificationAnimaliaColeopteraStaphylinidae

Li & Zhou sp. nov.

7AC818A4-FB8E-50A2-84CD-99F1B8C567DF

http://zoobank.org/2ACAA0B6-4CD7-47E4-AF05-334C7E3D8FF5

Fig. 1

#### Type specimens.

***Holotype*:** ♂, China: Gansu Province, Lanzhou City, Shifogou National Forest Park, Shifogou (石佛沟), 17.V. 2015, coll. Meng Wang (IZCAS). ***Paratypes***: 4 ♂♂, 6 ♀♀, same data as holotype (IZCAS).

**Figure 1.**
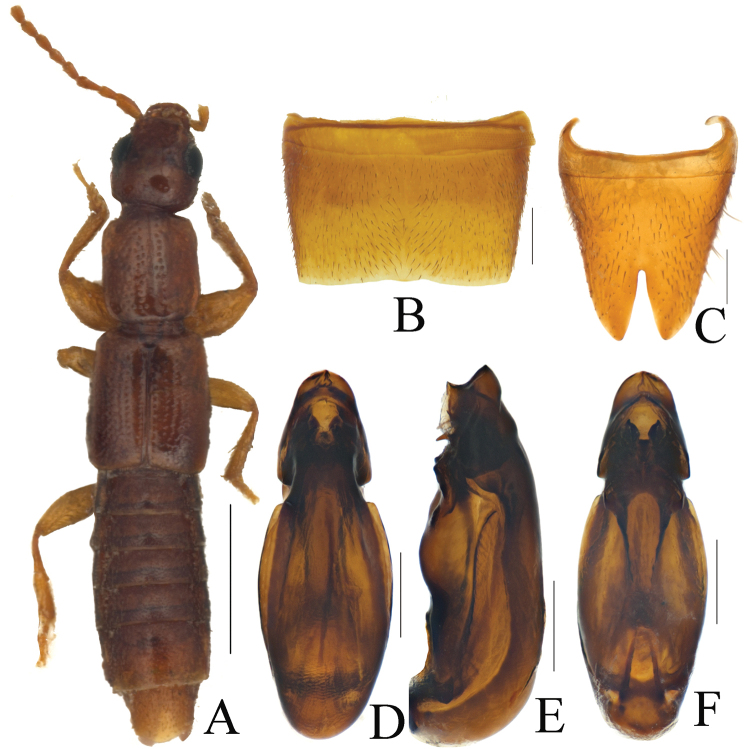
*Pseudolathra gansuensis* Li & Zhou, sp. nov., morphology **A** habitus **B** male sternite VII **C** male sternite VIII **D** aedeagus, dorsal view **E** aedeagus, lateral view **F** aedeagus, ventral view. Scale bars: 1.0 mm (**A**); 0.2 mm (**B–G**).

#### Description.

BL: 4.4–4.6 mm; FL: 2.4–2.5 mm. HL: 0.67 mm; HW: 0.62 mm; PL: 0.73 mm; PW: 0.64mm; EL: 0.93 mm; EW: 0.81 mm; EYL: 0.21mm; POL: 0.25mm.

***Body*** (Fig. 1A) elongate, brownish yellow; legs and antennae pale brown.

***Head*** (Fig. 1A) nearly square, slightly longer than wide and about 1.08 times as long as wide; punctures on vertex of head sparse and coarse, dense around eyes, intervals between punctures larger than diameter of a puncture; eyes big and slightly protruding, postocular portion approximately 0.73–0.76 times as long as eye length.

***Pronotum*** (Fig. 1A) oblong, slightly elongated, 1.14–1.16 times as long as wide, longer and broader than head. Anterior angles visible and posterior angles rounded, both sides straight. Longitudinal midline portion impunctate, both sides with dense and large punctures much denser and coarser than those of head and arranged in two compact rows generally; interstices with fine microsculpture.

***Elytra*** parallel-sided, longer than wide, longer than pronotum; punctures on surface arranged in 7 series in dorsal view; interstices without microsculpture. Hind wings fully developed.

***Abdomen*** approximately as broad as elytra, wider than head or pronotum; punctures very fine and dense; interstices with microsculpture; posterior margin of tergite VII with palisade fringe.

**Male. *Sternite VII*** (Fig. 1B) with a slight protrusion in middle, both sides of which slightly notched and surface with short hair slope to middle. Sternite VIII (Fig. 1C) with posterior excision narrow and deep, not quite reaching middle of sternite.

***Aedeagus*** (Fig. 1D–F), AEL= 0.91–0.93 mm long, length/width = 3.29. Dorsal plate fused with median lobe. Ventral process strongly sclerotized and curved ventrally (Fig. 1D–E). Middle lobe with apex round in dorsal or ventral view and internal sac with several strongly sclerotized and acute structures.

#### Distribution and remarks.

The species is known only from Gansu Province and the specimens were collected by light traps.

#### Comparative notes.

**Figure 2.**
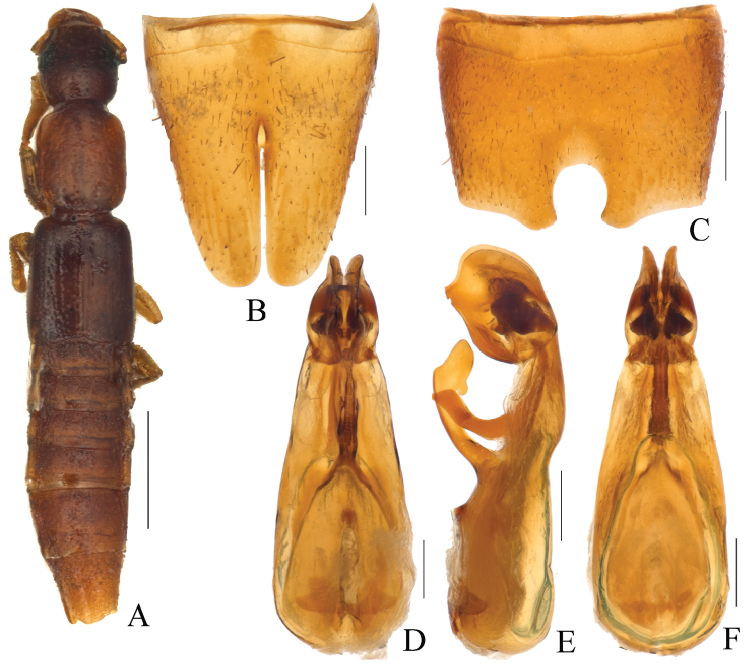
*Pseudolathra assingi* Li & Zhou, sp. nov., morphology **A** forebody **B** male sternite VII **C** male sternite VIII **D** aedeagus, ventral view **E** aedeagus, lateral view **F** aedeagus, dorsal view. Scale bars: 1.0 mm (**A**); 0.2 mm (**B–G**).

The new species is similar to *P. assingi* sp. nov. in habitus, but it can be distinguished from the latter by the deep notch in male sternites VII–VIII (Figs 1B, C, 2B, C), and the characteristics of the median lobe and interior armatures of the aedeagus are distinctly different (Fig. 1E–G).

The new species has a very similar aedeagus to *P. pulchella* (Kraatz, 1859), whereas the ventral protrusions of the median lobe are thinner than in the latter species. On the other hand, the middle notch of sternite VIII is distinctly deeper and narrower than in *P. pulchella* (Fig. 1B–C; Assing 2012: 315: figs 33–34; 321: 38–39).

#### Etymology.

The specific epithet is derived from the type locality, Gansu Province in Northwest China.

### Pseudolathra assingi

Taxon classificationAnimaliaColeopteraStaphylinidae

Li & Zhou sp. nov.

F4CC1484-BC9D-5804-A9DD-AF14C9001467

http://zoobank.org/7074F6B1-BB93-4C58-A4D4-07DBE8C1829E

Fig. 2

#### Type specimens.

***Holotype*:** ♂, China: Gansu Province, Lanzhou City, Shifogou National Forest Park, Shifogou (石佛沟), 17.V. 2015, coll. Meng Wang (IZCAS). ***Paratypes***: 1 ♂, 5 ♀♀, same data as holotype (IZCAS).

#### Description.

BL: 5.4–5.7 mm; FL: 2.8–3.1 mm. HL: 0.72 mm; HW: 0.72 mm; PL: 0.93 mm; PW: 0.78 mm; EL: 1.12 mm; EW: 0.93 mm; EYL: 0.29 mm; POL: 0.29 mm.

***Body*** (Fig. 1A) elongate, brownish yellow; legs and antennae straw yellow.

***Head*** (Fig. 1A) round, slightly wider than long and about 1.08 times as long as wide; punctures on vertex of head and around the eyes dense and coarse, with intervals between punctures as large as the diameter of a puncture; eyes big and flat, postocular approximately as long as eye.

***Pronotum*** (Fig. 1A) oblong, slightly elongated, 1.09–1.11 times as long as wide, 1.28–1.30 times as long and 1.07–1.09 times as broad as head; both anterior angles and posterior angles rounded, sides straight. Longitudinal midline portion impunctate; punctures on each side dense and larger than those of head and generally arranged in rows; interstices glossy with fine microsculpture.

***Elytra*** parallel-sided, longer than wide, slightly longer than pronotum; punctures on surface arranged in 5 series in dorsal view; interstices glossy with fine microsculpture. Hind wings fully developed.

***Abdomen*** approximately as broad as elytra, wider than head or pronotum; puntures of posterior tergites very fine and dense, whereas basal area with punctures larger than the former; interstices with fine microsculpture; posterior margin of tergite VII with palisade fringe.

**Male. *Sternite VII*** (Fig. 1B) with a round notch in middle, prominent on both sides. Sternite VIII (Fig. 1C) with posterior excision narrow and deep and about 2/3 length of this sternite, basal part with a half-fusiform depression.

***Aedeagus*** (Fig. 2D–F). AEL= 1.17 mm, length/width = 2.8. Dorsal plate fused with median lobe. Ventral process with a strongly sclerotized structure, curved ventrally and with apex irregularly formed (Fig. 2D–E). Middle lobe narrow in middle and bilobed posteriorly. Internal sac with several strongly sclerotized structures inside.

#### Distribution and remarks.

The species is known only from Gansu Province and the specimens were collected by light traps.

#### Comparative notes.

In addition to being similar to the previous species as described above, *P. assingi* sp. nov. also closely resembles *P. glabra* Peng, Li & Zhao, 2014, with differences as follows: 1) the former species has black head and elytra, whereas the latter one has brown head and elytra; 2) the notch of sternite VII, the ventral process, the hooks and the internal sac of the aedeagus differ significantly between the two species (Fig. 2E–G; Peng et al. 2014: 598, fig. 1B, D–E).

#### Etymology.

The specific epithet is from the given name of entomologist Dr. Volker Assing, in recognition of his great scientific contributions to the Chinese fauna of the genus *Pseudolathra*.

## Supplementary Material

XML Treatment for Pseudolathra gansuensis

XML Treatment for Pseudolathra assingi
